# Elevated Cardiac Troponin I following Asymptomatic Intradialytic Hypotension: A Pilot Study with a 2-Year Follow-Up

**DOI:** 10.1155/2022/4214429

**Published:** 2022-08-01

**Authors:** Toktam Alirezaei, Mir Jafar Jebreil Moosavi, Rana Irilouzadian, Elahe Taziki

**Affiliations:** ^1^Clinical Research Development Unit of Shohada-e Tajrish Hospital, Shahid Beheshti University of Medical Sciences, Tehran, Iran; ^2^School of Medicine, Shahid Beheshti University of Medical Sciences, Tehran, Iran

## Abstract

**Background:**

Intradialytic hypotension (IDH) has been recognized as a serious and frequent complication during hemodialysis (HD) of end-stage renal disease (ESRD) patients, but the effect of asymptomatic IDH on cardiac troponin I (cTnI) levels is not definitively elucidated.

**Methods:**

70 asymptomatic HD patients with negative predialysis cTnI were included. They were on maintenance HD thrice weekly. All patients were monitored during the HD session for hemodynamic changes and symptoms related to IDH. Patients were followed for two years, and their outcomes are noted as an acute coronary syndrome (ACS), cardiac death, no ACS, noncardiac death, and kidney transplant.

**Results:**

Compared with the baseline blood pressure values, there was a drop in systolic blood pressure for all subjects, but according to the 2007 European Best Practice Guidelines on hemodynamic instability, asymptomatic IDH was defined in 27 (38.6%) patients. The results demonstrated a significant correlation (*r* = 0.492) (*p* < 0.05) between asymptomatic IDH and elevated postdialysis levels of cTnI. In 2-year follow-up of patients, ACS and cardiac death happened more in patients with elevated cTnI.

**Conclusion:**

The results of our study suggest that asymptomatic IDH affects cTnI levels. Given that cTnI is a marker of myocardial damage and a predictor of cardiovascular mortality in ESRD patients, these findings recommend that considering the asymptomatic decrease in blood pressure levels during HD is very important and critical.

## 1. Introduction

Intradialytic hypotension (IDH), the most frequent hemodynamic side effect during dialysis, remained a challenge in hemodialysis (HD) settings and affects 22–30% of all HD patients [1, 2]. HD can precipitate myocardial ischemia. Cardiovascular diseases are the cause of more than half of mortality cases in HD patients [3–5]. However, relatively little attention has been paid to silent ischemia during dialysis. Asymptomatic (silent) myocardial ischemia is a common event in HD patients which is found in more than 33% of these patients [6].

Cardiac troponin I and T (cTnI and cTnT, respectively) are the diagnostic biomarkers for myocardial injury [7]. Elevation of cTnT occurs in a variety of nonischemic conditions including renal failure, sepsis, heart failure, and pulmonary edema [8]. Contrarily, cTnI is one of the most sensitive and specific markers for myocardial cell injury and exhibits a higher specificity than the cardiac isoenzyme creatine kinase (CK-MB) and greater sensitivity and specificity than cTnT. Even silent myocardial damage can be identified by elevated cTnI [9–12]. The specificity of cTnI in patients undergoing chronic dialysis without acute coronary syndrome is nearly 100% [13, 14]. According to a study by Roppolo et al., the levels of cTnI in asymptomatic HD patients had a nearly 100% positive prediction rate of cardiac events. [15].

IDH has been recognized as a predictor of cardiovascular mortality, and severe IDH may result in acute cardiac complications such as myocardial infarction [16, 17]. It is suggested that even an asymptomatic decrease in blood pressure (BP) may have adverse effects. Repeated episodes of asymptomatic IDH can lead to myocardial damage. [18].

In this study, we recorded the hemodynamic status and cTnI levels in chronic HD patients with normal baseline troponin (levels<0.29 ng/ml) during HD and followed up with them after 2 years to determine their long-term prognostic value for cardiovascular events.

## 2. Materials and Methods

### 2.1. Participants

This prospective study was conducted on patients older than 35 years and younger than 70 years of age on chronic HD treatment for at least more than a month and cTnI levels of less than 0.29 ng/ml before starting HD from November 2018 to January 2020. Patients with HD duration less than 3 hours as well as those with cardiac diseases such as ischemic heart disease (IHD), valvular heart disease, left ventricular systolic dysfunction, or heart failure (EF<50%) were excluded from the study. As a result, 70 out of 208 end-stage renal disease (ESRD) patients who referred to the dialysis department of Shohada-e Tajrish hospital, Tehran, Iran, were included in the study and were observed in a cross-sectional manner for about 4-5 hours during HD sessions. Patients with diabetes mellitus are defined based on American Diabetes Association guidelines [19] and/or those who used antidiabetic agents. Hypertension is defined based on 2018 European Society of Cardiology/European Society of Hypertension guidelines [20] and/or patients who took any antihypertensive drugs.

### 2.2. Materials

The primary objectives were to evaluate the prevalence of HD-induced hemodynamic changes in a standard HD population and its relation to elevated cTnI. A secondary objective was a 2-year follow-up of the patients for cardiac and noncardiac outcomes.

IDH definition: blood pressure (BP) was measured pre- and postdialysis, as well as every 15 minutes using an automated digital oscillometric device (model: SAADAT, ALBORZ, B9, Iran). Hemodynamic changes or IDH were defined according to the 2007 European Best Practice Guidelines (EBPG) as a drop in systolic blood pressure (SBP) of more than 20 mmHg at each time point compared to the predialysis reading. Reference to the 2007 EBPG on hemodynamic instability: a decrease in systolic blood pressure (SBP) ≥20 mmHg or in mean arterial pressure (MAP) ≥10 mmHg is associated with a clinical event and the need for nursing intervention. [21].

### 2.3. Measurement of cTnI

A single measurement of cardiac biomarkers was performed instead of using the mean/median of multiple measurements [22]. Nonfasting cTnI concentrations were measured on an immunoassay system analyzer (SIEMENS IMMULITE 2000XPI) using the manufacturer's assay kits. The reference ranges for cTnI (at the 99 percentile) were a concentration of 0.29 ng/ml. cTnI cutoff values were <0.29 ng/ml, 0.29–0.99 ng/ml, 1–1.99 ng/ml, 2–2.99 ng/ml, 3-4 ng/ml, and > 4 ng/ml.

### 2.4. Procedure

Written informed consent has been acquired from the patients, and the study has been approved by the ethics review board of Shahid Beheshti University of Medical Sciences (ethical code: IR.SBMU.RETECH.REC.1397.237). Blood samples were collected from patients before and after HD sessions. The collected samples were then centrifuged for 4 minutes in the laboratory of the same hospital with analyses being performed within 2 hours after the collection of samples. An electrocardiogram (ECG) was obtained from all of the patients. cTnI levels were measured in blood samples at a single point in time. Laboratory personnel was involved in a double-blinded procedure when handling patients' data to reduce interassay variability and bias. Patients were followed up for two years, and their outcomes are noted as an acute coronary syndrome (ACS), cardiac death, no ACS, noncardiac death, and kidney transplant.

### 2.5. Statistical Analysis

Statistical analyses were performed using Statistical Package for Social Science (SPSS) for Windows version 16.0 (SPSS Inc. Chicago, IL). The normality test was performed using the Shapiro–Wilk test. Continuous variables were expressed as mean ± standard deviation and categorical variables as numbers and percentages. Pearson chi-square test was used. Also, a paired sample *t*-test was used to evaluate the difference in hemodynamic changes according to cTnI results, with *P* value <0.05 being considered statistically significant. Spearman correlation was used to evaluate the correlation between 2-year outcomes and IDH and cTnI changes.

## 3. Results

A total of 70 patients were enrolled in the study, and their baseline characteristics and dialysis-related factors are shown in Table 1. The mean age was 58.4 ± 12 years old, and 34 (48.6%) patients were male. Diabetes mellitus and hypertension were present in 10 (14.3%) and 18 (25.7%) patients, respectively. Hemodialytic parameters included all patients being dialyzed 3 times a week. An arteriovenous fistula was utilized for vascular access in 31 patients (44.3%), whereas a central venous catheter was utilized for the remaining 39 (55.7%) patients, comprising 34 (48.6%) with a permanent catheter and 5 (97.1%) with double lumen dialysis catheter. While there was no significant difference in ultrafiltration rate between the patients with IDH and those with no IDH, blood flow had a significant difference between these two groups (Table 1).

**Figure 1:**
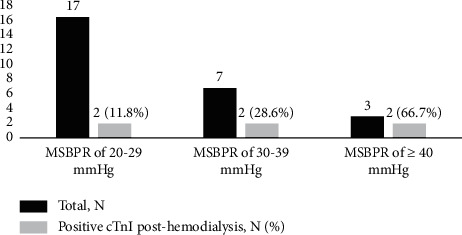
Association between maximum systolic blood pressure reduction (MSPR) and positive cTnI according to EBPG definition.

All ESRD patients revealed a drop in SBP in the range of less than 10 mmHg to more than 40 mmHg during HD sessions but an SBP drop of more than 20 mm Hg, as regarded in the 2007 EBPG definition of intradialytic hypotension (IDH) was found in 27 patients (38.6%) during HD sessions. cTnI levels were measured before the beginning of HD and after the end of HD. The patients with elevated predialysis cTnI were excluded from the study. cTnI elevation after HD occurred in 6 patients (8.6%), while no episode of intradialytic chest pain or dialysis-induced dyspnea, dizziness, or diaphoresis was observed. None of the patients needed any nursing intervention during the HD. ECGs of all of the patients showed no significant ST-T changes. All of the patients who had elevated cTnI also experienced IDH. There was a significant correlation between hemodynamic changes and elevated cTnI levels (*p* < 0.003, *r* = 0.345) (Table 2).  Table 3 and Figure 1 show the association between maximum SBP reduction (MSBPR) during HD and elevation in cTnI levels in our HD patients. There was a significant correlation between the decrease in SBP during HD and the increase in cTnI (*p* < 0.001, *r* = 0492). Elevation in cTnI is more likely when SBP is more reduced; for instance, when SBP reduction was less than 20 mmHg, cTnI levels were negative; in 17 patients with MSBPR of 20–29 mmHg, only in 2 patients (11.8%), cTnI levels were elevated, but when MSBPR was more than 40 mmHg in 3 patients, cTnI levels were elevated in 2 of them (66.7%).

### 3.1. Follow-Up

The 2-year follow-up of the patients showed that most of the patients with negative cTnI did not develop ACS, and mortality was mostly due to noncardiac causes which COVID-19 had the most prevalent cause. Patients with positive postdialysis cTnI whom they all had MSBPR more than 20 mmHg experienced cardiac death (33.6%) and ACS (16.6%) more than the patients with negative postdialysis cTnI. However, the analysis had a *p* value of >0.05 which may be due to a low number of patients with elevated cTnI (Table 4).

## 4. Discussion

Studies showed that ultrafiltration during HD prompts IDH which is strongly associated with mortality. IDH results in decreased blood flow to the myocardium and, as a result, myocardial stunning [23]. HD-induced myocardial stunning was not only associated with increased mortality but also with the first cardiovascular event latency. IDH, even asymptomatic, is an independent risk factor for mortality and cardiovascular diseases in HD patients [24–26]. A significant relationship has been found between reduced SBP and increased levels of cTnI during the HD procedure, a finding that represents a possible therapeutic target. Such treatments are involved in the improvement of both intradialytic hemodynamics and plasma concentration of biochemical markers of myocardial injury. [24].

Elevated levels of cTnI are commonly observed in ESRD patients regardless of the presentation of acute symptoms and signs of cardiac disease. Since cTnI levels are considered the reference biomarker for the diagnosis of acute myocardial infarction, these persistently elevated levels may pose diagnostic challenges in ESRD patients. In a study by Castini et al. on 30 HD patients, individual intradialytic changes in cTnI were evaluated in three different HD sessions a week. Only 6 episodes of IDH were noted which were not associated with cTnI elevation. They also demonstrated that HD did not significantly affect cTnI levels [27]. However, these studies had a small population size, different definitions of IDH, and a low SBP reduction during HD.

In a study by Mavrakanas et al., high ultrafiltration was correlated with elevated cTnI levels but not IDH. However, in our study, we observed a significant correlation between IDH and an increase in cTnI levels. This may result from their significantly different definition for IDH, which is a reduction of 50 mmHg in SBP, whereas we defined a reduction of more than 20 mmHg or higher as significant according to EBPG definition [28] Tarapan et al. showed that high-sensitivity cTnI levels are higher in ESRD patients on HD than in the non-CKD control group. [29].

In a study by Hung et al. in 70 HD patients, they found that patients with symptomatic IDH had a significant increase in cTnI, and the patients with cTnI ≥0.20 ng/ml developed cardiovascular diseases and even death [30].

The first aim of our study was to evaluate the effect of hemodynamic changes including SBP reduction on cTnI levels during HD sessions in a group of stable ESRD patients who are asymptomatic for chest pain and other symptoms that are indicative of cardiac ischemic. To investigate intradialytic changes, we compared pre-HD cTnI levels with post-HD levels on the same day. To our knowledge, this is the first study that evaluated intradialytic hemodynamic and cTnI changes in a single-day time frame, while other studies have compared pre-HD cTnI values on a weekly or monthly time frame.

In our study, we observed elevated cTnI levels in 8.6% of asymptomatic and clinically stable ESRD patients. All patients were on maintenance HD three times a week with a median dialysis age of 35 months. The prevalence of DM and HTN were 14% and 25%, respectively. Analyzing cTnI changes with the cutoff value of 0.29 ng/ml showed a significant increase after HD in 6 patients who also had IDH. As previously mentioned, our patients were asymptomatic during the HD procedure, and no significant chest pains were observed. Finally, no correlation was found between intradialytic heart rate changes, age, gender, ultrafiltration rate or EKG abnormalities, and elevated cTnI levels.

Previous studies that evaluated the association of cardiac troponins with mortality in asymptomatic HD patients utilized cTnT instead of cTnI. Also, they mostly used a less sensitive assay with relatively high cutoff values. In our study, we focused on high-sensitivity cTnI and the additional prognostic value of moderate elevation of cTnI. Another strength of our study is that we also followed up our 70 patients for 2 years to verify the association between cTnI elevation and future cardiac events. The 2-year follow-up of the patients showed that most of the patients with negative cTnI did not develop ACS and mortality was mostly due to noncardiac causes which COVID-19 had the most prevalent cause. In HD patients with elevated cTnI whom they all had MSBPR more than 20 mmHg, ACS and cardiac death were seen more than in those without elevated cTnI. Our study has some limitations. First, our study was a single-center study and did not have a big study population; however, it was not different from other studies. Also, there is a possibility of false-negative results in our study because our patients were not hospitalized, and post-HD cTnI was checked at the end of the HD session. By taking into consideration the fact that cTnI will be positive about 3 hours after cardiac ischemia, it is possible to conclude that may be some cTnI had not been positive at the end of HD. Therefore, we suggest a study on the association between IDH and cardiac dysfunction in ESRD patients.

## 5. Conclusion

From our results, it can be concluded that there is a significant relationship between SBP reduction during HD and an increase in cTnI levels in ESRD patients. This study supports the disputation that subclinical myocardial injury is commonly provoked by HD, wherein such episodes of damage can be associated with cardiac dysfunction, additional cardiac events, and reduced patient life expectancy in long term. It is a given possibility that treating IDH, even asymptomatic ones, may lead to a decrease in cardiac mortality in HD patients.

## Figures and Tables

**Table 1:** Baseline characteristics of the study group.

	All patients (*N* = 70)	Non-IDH patients (*N* = 43)	IDH patients (*N* = 27)		*P* value
			Negative cTnI (*N* = 21)	Positive cTnI (*N* = 6)	
Age (years), mean ± SD	58.4 ± 12	57.6 ± 13	61.6 ± 10	53.3 ± 13	0.273
Male, *N* (%)	34 (48)	18 (42)	12 (57)	4 (67)	0.336
Female, *N* (%)	36 (52)	25 (58)	9 (43)	2 (33)	0.336

Dialytic age (months), mean ± SD	35.6 ± 33	34.9 ± 35	38.3 ± 30	30.8 ± 33	0.872
BMI (kg/m^2^), mean ± SD	25.7 ± 5	25.2 ± 5	26.8 ± 4	26.1 ± 5	0.493
BF (mL/min), median (IQR)	252.2 (26)	246.5 (27)	257.1 (14)	276.6 (38)	0.018
UF (mL/h), mean ± SD	566.1 ± 134.1	546.5 ± 136.4	571.4 ± 101.3	687.5 ± 172.3	0.051
BUN (mg/dL), mean ± SD	53.0 ± 19	54.3 ± 20	54.3 ± 19	38.6 ± 8	0.174
Cr (mg/dL), mean ± SD	6.6 ± 2	6.4 ± 2	6.8 ± 3	7.1 ± 2	0.678
Albumin (mg/dL), mean ± SD	3.5 ± 1	3.44 ± 0.5	3.7 ± 2	3.8 ± 0.7	0.510
ESR (mm/h), mean ± SD	33.2 ± 27	36.5 ± 31	27.4 ± 20	29.6 ± 16	0.441
CRP (mg/L), mean ± SD	16.3 ± 20	19.8 ± 23	11.7 ± 13	8.0 ± 6	0.189
Ferritin (mcg/L), mean ± SD	454.8 ± 322	500.7 ± 369	366.7 ± 209	434.1 ± 250	0.295
PTH (pg/mL), mean ± SD	322.9 ± 314	320.8 ± 322	311.6 ± 293	377.3 ± 370	0.903
Vitamin D (pg/mL), mean ± SD	26.8 ± 16	25.5 ± 16	27.6 ± 17	33.3 ± 15	0.546
MPV (fL), mean ± SD	8.8 ± 1	8.7 ± 1.5	8.9 ± 2	9.8 ± 1.5	0.301
Hb (g/dL), mean ± SD	11.2 ± 1.4	11.2 ± 1.3	10.9 ± 1.4	12.2 ± 1.7	0.129
HCT (%), mean ± SD	34.1 ± 3.5	34.3 ± 3.3	34.2 ± 3.7	34.8 ± 4.9	0.861

ESRD etiology, N (%)					0.185
DM	10 (14.3)	7 (16.2)	3 (14.3)	0	
HTN	18 (25.7)	9 (20.9)	7 (33.3)	2 (33.3)	
ADPKD	2 (2.8)	0	2 (9.5)	0	
Glomerulonephritis	2 (2.8)	2 (4.6)	0	0	
Obstructive	8 (11.4)	6 (14.0)	2 (9.5)	0	
Others	7 (10.0)	5 (11.6)	2 (9.5)	0	
DM and HTN	23 (47.1)	14 (32.5)	5 (23.8)	4 (66.7)	

ADPKD, autosomal dominant polycystic kidney disease; BMI, body mass index; BF, blood flow rate in dialysis; BUN, blood urea nitrogen; Cr, creatinine; CRP, C-reactive protein; cTnI, cardiac troponin I; DM, diabetes mellitus; ESR, erythrocyte sedimentation rate; ESRD, end-stage renal disease; Hb, hemoglobin; HCT, hematocrit; HTN, hypertension; IDH, intradialytic hypotension; MPV, mean platelet volume; PTH, parathyroid hormone; SD, standard deviation; UF, ultrafiltration rate.

**Table 2:** Predialysis and postdialysis hemodynamic status.

	All patients (*N* = 70)	Non-IDH patients (*N* = 43)	IDH patients (*N* = 27)		*P* value
			Negative cTnI (*N* = 21)	Positive cTnI (*N* = 6)	
Systolic blood pressure (mmHg)					
Predialysis, mean ± SD	133.4 ± 24.1	126.9 ± 21.2	144.1 ± 24.9	142.8 ± 28.3	0.014
Postdialysis, mean ± SD	119.0 ± 22.3	117.6 ± 20.2	122.9 ± 25.9	114.7 ± 24.8	0.6
Diastolic blood pressure (mmHg)					
Predialysis, mean ± SD	78.4 ± 12.9	75.9 ± 11.6	82.3 ± 12.0	82.7 ± 21.2	0.12
Postdialysis, mean ± SD	74.9 ± 11.3	73.9 ± 9.7	76.4 ± 12.1	76.5 ± 19.6	0.667
Heart rate (bpm)					
Predialysis, mean ± SD	78.8 ± 8.3	78.3 ± 5.8	79.1 ± 9.4	81.8 ± 17.3	0.611
Postdialysis, mean ± SD	82.7 ± 8.5	81.9 ± 5.7	83.4 ± 10.8	85.3 ± 15.7	0.597
ECG abnormalities	0	0	0	0	

Bpm, beats per minute; cTnI, cardiac troponin I; DBP, diastolic blood pressure; ECG, electrocardiogram; HR, heart rate; IDH, intradialytic hypotension; SBP, systolic blood pressure; SD, standard deviation.

**Table 3:** Distribution of patients based on maximum systolic blood pressure reduction (MSBPR) and cTnI.

	MSBPR				
	Less than 20 mmHg	20–29 mmHg	30–39 mmHg	More than 40 mmHg	Total
Negative cTnI, *n* (%)	43 (61.4%)	15 (21.4%)	5 (7.1%)	1 (1.4%)	64 (91.4%)
Positive cTnI, *n* (%)	0	2 (2.9%)	2 (2.9%)	2 (2.9%)	6 (8.6%)
0.29−0.99	0	0	1	1	2 (2.9%)
1−1.99	0	0	1	0	1 (1.4%)
2−2.99	0	1	0	0	1 (1.4%)
3−3.99	0	1	0	0	1 (1.4%)
4−4.99	0	0	0	0	0
5−5.99	0	0	0	1	1 (1.4%)
Total, *N* (%)	43 (61.4%)	17 (24.3%)	7 (10.0%)	3 (4.3%)	70 (100.0%)

CTnI, cardiac troponin I; MSBPR, maximum systolic blood pressure reduction. ^*∗*^Linear by linear association and chi-square tests showed a *p* value of <0.05.

**Table 4:** Outcomes in all patients after 2-year follow-up.

	MSBPR <20 mmHg and postdialysis negative cTnI (*N* = 43)	MSBPR >20 mmHg		*P* value
		Postdialysis positive cTnI (*N* = 6)	Postdialysis negative cTnI (*N* = 21)	
Cardiac death	3 (7%)	2 (33.6%)	2 (9.5%)	0.510
Noncardiac death	13 (30%)	1 (16.6%)	8 (38.1%)	
COVID-19	10	1	7	
Sepsis	1	0	1	
Influenza	1	0	0	
Trauma	1	0	0	
ACS	3 (7%)	1 (16.6%)	2 (9.5%)	
No ACS	22 (51%)	1 (16.6%)	8 (38.1%)	
Kidney transplant	2 (5%)	1 (16.6%)	1 (4.8%)	

ACS, acute coronary syndrome; COVID-19, corona virus disease 2019; cTnI, cardiac troponin I; MSBPR, maximum systolic blood pressure reduction.

## Data Availability

The data that support the findings of this study are available upon request from the corresponding author.
